# A pilot study to assess the origin of the spectral power increases of heart rate variability associated with transient changes in the R‐R interval

**DOI:** 10.14814/phy2.15907

**Published:** 2024-01-16

**Authors:** Satoshi Mitsuyama, Teruhiko Sakamoto, Toru Nagasawa, Kazuomi Kario, Seiji Ozawa

**Affiliations:** ^1^ Department of Healthcare Informatics Takasaki University of Health and Welfare Takasaki Gunma Japan; ^2^ Division of Cardiovascular Medicine, Department of Medicine Jichi Medical University School of Medicine Tochigi Japan

**Keywords:** arterial baroreceptor reflex, frequency resolution, heart rate variability, spectral analysis, time resolution, transient RRI increase

## Abstract

Spectral analysis of heart rate variability (HRV) is used to assess cardiovascular autonomic function. In the power density spectrum calculated from a time series of the R‐R interval (RRI), three main components are distinguished: very‐low‐frequency (VLF; 0.003–0.04 Hz), low‐frequency (LF; 0.04–0.15 Hz), and high‐frequency (HF; 0.15–0.4 Hz) components. However, the physiological correlates of these frequency components have yet to be determined. In this study, we conducted spectral analysis of data segments of various lengths (5, 30, 100, and 200 s) of the RRI time series during active standing. Because of the trade‐off relationship between time and frequency resolution, the analysis of the RRI data segment shorter than 30 s was needed to identify the temporal relationships between individual transient increases in RRI and the resulting spectral power changes. In contrast, the segment of 200 s was needed to properly evaluate the magnitude of the increase in the VLF power. The results showed that a transient increase in the RRI was tightly associated with simultaneous increases in the powers of the VLF, LF, and HF components. We further found that the simultaneous power increases in these three components were caused by the arterial baroreceptor reflex responding to rapid blood pressure rise.

## 1 INTRODUCTION

Heart rate variability (HRV) is the variability in the time between successive heartbeats. Spectral analysis of HRV is widely used as a noninvasive method to assess cardiovascular autonomic function (Akselrod et al., 1981; Billman, 2011; Hayano & Yuda, 2019; Pagani et al., 1986; Task Force of the European Society of Cardiology and the North American Society of Pacing and Electrophysiology, 1996). In the spectrum calculated from a beat‐to‐beat time series of the R‐R interval (RRI), three main components are distinguished: very‐low‐frequency (VLF; 0.003–0.04 Hz), low‐frequency (LF; 0.04–0.15 Hz), and high‐frequency (HF; 0.15–0.4 Hz) components. Although it is generally accepted that the HF power reflects modulation of heart rate mediated by cardiac parasympathetic (vagal) nerve activity, the origin and physiological correlate of the LF component are controversial, and those of the VLF component are mainly unknown.

In a previous study, we attempted to clarify the temporal relationship between transient RRI and LF power changes. We consecutively conducted an ultra‐short‐term spectral analysis of 5‐ and 25‐s data segments of the RRI time series with Fast Fourier Transform (FFT) (Yokobori et al., 2023). We found that a transient RRI increase that occurred occasionally during active standing was associated with an increase in LF power, which was also linked to a small increase in HF power. However, the following three problems remained unsettled in the previous work. First, short‐term RRI data segment analysis reduces the frequency resolution and makes it difficult to detect changes in the magnitude of the power of low‐frequency signals. To overcome this trade‐off relationship between time and frequency resolutions, we have to optimize the length of the data segments to be analyzed depending on the specific requirements in the signal analysis (Dorran, 2021; Smith, 1999). Second, we found that the LF power increase upon the transient RRI increase was also linked to the small HF power increase. This finding suggested that the transient RRI increase would increase power in the LF and other frequency ranges. In particular, the contribution of the power increase in the VLF range, which had been neglected in the previous analysis, should be examined. Third, we needed to identify the origin and physiological correlate of the power increases in various frequency ranges linked to the transient increase in RRI. Several groups have proposed that LF power fluctuation is accounted for by the arterial baroreceptor reflex (Cevese et al., 2001; Goldstein et al., 2011; Grasso et al., 1997; Moak et al., 2007; Rahman et al., 2011; Sleigh et al., 1995). This notion should be verified more directly.

In this study, we attempted to solve the above problems. We reanalyzed some of the data obtained in the previous work and added new experiments. We found that the transient increase in RRI that occurred occasionally during standing was associated with simultaneous increases in the powers of the VLF, LF, and HF components. We also obtained direct evidence that the arterial baroreceptor reflex generated these changes in response to a rapid increase in arterial blood pressure.

## 2 MATERIALS AND METHODS

### 2.1 Subjects

We reanalyzed the data from five subjects in the previous study (two males and three females, 20–22 years of age when the data were obtained) (Yokobori et al., 2023). The other eight subjects (three males and five females, 20–25 years of age) participated in the experiments to study the relationship between HRV and changes in beat‐to‐beat arterial blood pressure. All participants were in good health without cardiovascular and respiratory diseases and were not taking subscription medications regularly. They provided their written informed consent to participate in the study protocol, which the Ethical Committee of Takasaki University of Health and Welfare approved.

### 2.2 Instrumentation and data analysis of HRV

HRV was measured using a wearable electrocardiogram sensing device (myBeat; Union Tool, Co., Tokyo, Japan). This device was worn around the epigastrium on the subject's chest and detected the peak of the QRS complex to obtain the beat‐to‐beat time series of the RRI.

Because the RRI time series occasionally contained abnormal values caused by noise, the RRI value that deviated from the range between 300 and 2000 ms was omitted. In addition, it was omitted if the instantaneous heart rate was either 30 bpm greater or less than the average of the preceding eight values. Continuous RRI signal data as a function of time were obtained from discrete RRI event series using a spline interpolation method. They resampled at 100‐ms intervals for FFT processing.

We analyzed the time series of the RRI data obtained using the RRI Analyzer 2 software (Union Tool, Co.). Before FFT processing, Hann window processing was performed. Based on the power spectral density (PSD) of each RRI data segment, the VLF, LF, and HF powers were calculated by integrating from 0.003 to 0.04 Hz, from 0.04 to 0.15 Hz, and from 0.15 to 0.4 Hz, respectively.

For analyses of the RRI data sequence of various lengths, the time window for FFT processing was set to 204.8 s to ensure 0.0048 Hz frequency resolution, which required 2048 data points sampled at 10 Hz from the RRI data sequence. However, in this study, the duration of the RRI data segment was too short to give 2048 data points. Zero padding was adopted to overcome this problem. Although zero padding can improve the interpolation density in the frequency domain, it generates artifacts named sidelobes, which are additional peaks or ripples in the frequency spectrum outside the main lobe (Lyons, 2011; Smith III., 2007). Care was taken to evaluate the results obtained using zero padding (see Discussion).

### 2.3 Reanalysis of previous data

We addressed the first and second problems in the previous analysis described in the Introduction by reanalyzing 12 600‐s RRI time series data during standing obtained from five subjects in the previous study. To acquire spectral information on changes in the powers of the different frequency components linked to the transient RRI change, we adopted the time‐frequency analysis method based on the short time Fourier transform (STFT) (Jablonski & Dziedziech, 2022; Zhu et al., 2007). This method is widely used for time‐frequency representations of real‐world nonstationary signals, such as audio signals.

(1) Evaluation of the magnitude of increases in power associated with transient RRI change.

**FIGURE 1:**
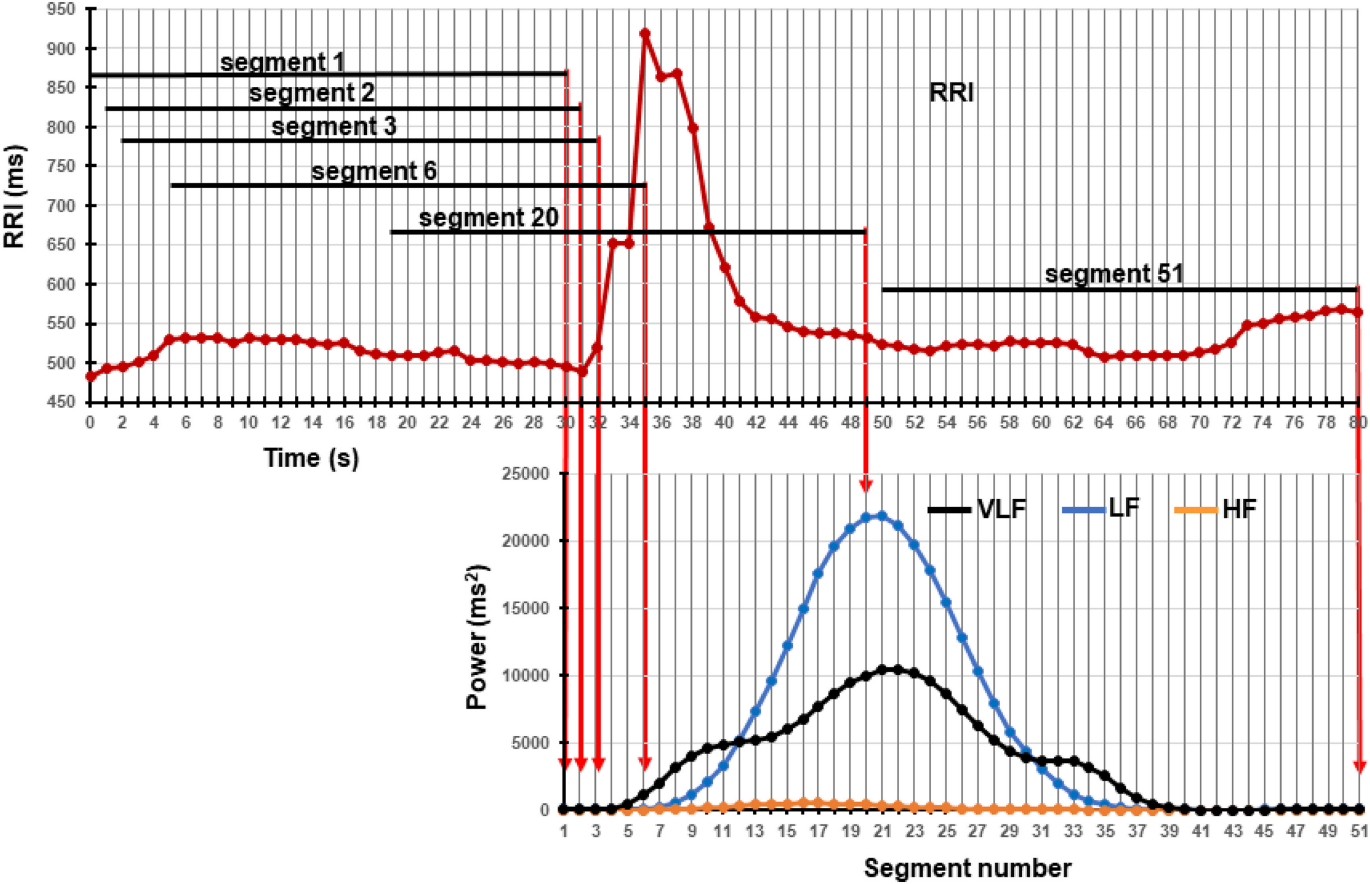
Example of evaluation of changes in powers of very‐low‐frequency (VLF), low‐frequency (LF), and high‐frequency (HF) components associated with a transient RRI increase during standing. In this case, the length of the data segment was set to 30 s. (top) RRI data sequence during the first 80 s of a 600‐s RRI time series data plotted at 1‐s intervals. From this sequence, 51 30‐s RRI data segments were made consecutively with a 1‐s shift. (bottom) Plots of the magnitudes of powers of three frequency components calculated in 51 30‐s RRI data segments. The magnitudes of the powers in each segment are plotted at the end of the segment, indicated by red down arrows.

In this study, we analyzed the RRI time series data during the 600‐s standing using RRI data segments of discrete length between 5 to 200 s. To avoid contamination by noise due to body movements and changes in RRI caused by an abrupt decrease in blood pressure upon active standing (Harms et al., 2021), the RRI time series data began 60 s after the postural change. To track the temporal relationship of the magnitude of power changes in each frequency component with the transient changes in RRI, we performed time‐frequency analysis for nonstationary signals by first setting the length of the data segment to 30 s. STFT of the 30‐s segment was performed consecutively with a 1‐s shift to obtain PSD, and the power magnitudes of VLF, LF, and HF components were calculated with a 1‐s shift from each PSD. Figure 1 illustrates an example to evaluate the VLF, LF, and HF power increases associated with a transient RRI increase in the first 80 s of a 600 s RRI time series data while standing. The RRI change is plotted at 1‐s intervals on the graph's top panel. We consecutively made 51 30‐s RRI data segments with a 1‐s shift and obtained the PDS within each segment by applying STFT. The magnitudes of the VLF, LF, and HF powers in each segment were then calculated and plotted on the bottom panel. We defined the means of the powers in the frequency ranges obtained from these 51 segments as the magnitudes of the VLF, LF, and HF powers (3565.8, 5538.1, and 130.5 ms^2^, respectively) in this short RRI time series. Extending this analysis to 600 s, we evaluated the power magnitudes in the VLF, LF, and HF ranges during 600 s as the means of each magnitude of 571 30‐s RRI data segments.

When the length of the data segment was set to 5, 100, and 200 s, we could obtain the magnitudes of the VLF, LF, and HF powers for 600 s as the means of each power of 596, 501, and 401 data segments, respectively. By estimating the effects of changing the length of the data segment on the mean values calculated above, we could estimate how the trade‐off relationship between time and frequency resolution occurred in our analysis.

(2) Selection of the RRI time series data for reanalysis.

In the previous analysis, we analyzed the 25‐s RRI data segments (Yokobori et al., 2023). To examine changes in LF and HF powers over the supine to standing postural change, we divided the RRI time series of each 600 s before and during standing into 24 25‐s segments and obtained the LF and HF powers in each segment. We then calculated the average value of each 24 segments before and during standing, and used the ratios of these averages (standing: supine ratios of the means of LF and HF powers) as indices of changes in the LF and HF powers associated with postural change. In the standing: supine ratios obtained in this way, the ratio of the HF power invariably decreased with a marked decrease in the magnitude of respiratory sinus arrhythmia (Berntson et al., 1993; Eckberg, 1983; Taylor et al., 1999). In contrast, the ratio of the LF power was widely distributed. We referred to the response pattern to standing as type 1 when the standing: supine ratio of the LF power was ≤1 and as type 2 when the ratio was >1. However, the RRI data segment of 25 s could be too short to estimate the magnitude of the powers of the lower frequency components correctly. Therefore, in the present trial, we calculated the values of the LF power from the PSDs of each 600 s before and during standing and used them for the classification of type 1 and type 2 cases. In this study, we reanalyzed the data obtained in the previous experiment by selecting six representative RRI time series data, each from the most typical type 1 and type 2 cases obtained from five subjects.

### 2.4 Simultaneous monitoring of changes in RRI and beat‐to‐beat finger arterial pressure

To identify a physiological correlate of the changes in the VLF, LF, and HF powers associated with the transient RRI increase, we measured beat‐to‐beat arterial blood pressure with a finger arterial blood pressure monitoring device (viewphii‐CBP, Socionext, Inc., Yokohama, Japan) during HRV measurement (Nakagawara & Yamakoshi, 2000; van Wijnen et al., 2017; Yamakoshi et al., 1980). Using this device, we continuously measured changes in arterial mean blood pressure (MBP). Also, we obtained a time series of the interbeat interval (IBI), which was similar to that of the RRI simultaneously recorded with the wearable electrocardiogram sensing device described above. The spectral analysis result on the time series of IBI was the same as that of RRI in all tested cases.

In this series of experiments, the eight subjects wearing both the RRI and continuous blood pressure measuring devices adopted a supine position for 6–12 min, then stood quietly and remained standing for 12 min. They performed this trial 1–3 times, and altogether 16 measurements were performed.

### 2.5 Statistical analysis

The data are presented as the mean ± standard deviation (SD). A statistical comparison between the two groups was performed using Student's or Weltch's *t*‐test.

## 3 RESULTS

### 3.1 Small increase in HF power linked to LF power increase associated with a transient increase in RRI

We previously found that the postural change from supine to standing always caused a marked decrease in the HF power, whereas it reduced the LF power in most cases but increased in a minority of cases (Yokobori et al., 2023). In the present study, we calculated the values of the LF power from the PSDs of each 600 s before and during standing. We referred to the response pattern to standing as type 1 when the standing: supine ratio of the LF power was ≤1 and as type 2 when the ratio was >1. In these analyses, the RRI time series data 60 s immediately before and after the postural change were omitted to avoid contamination by noise due to body movements and changes in RRI caused by an abrupt decrease in blood pressure upon standing. We selected each of the six RRI time series data of the most typical type 1 and type 2 cases obtained in the previous experiment and reanalyzed them in this way. The standing: supine ratio of the LF powers in type 1 cases ranged from 0.18 to 0.47 (mean 0.31 ± 0.12, *n* = 6), and that in type 2 cases ranged from 2.05 to 5.14 (mean 2.73 ± 1.20, *n* = 6).

**FIGURE 2:**
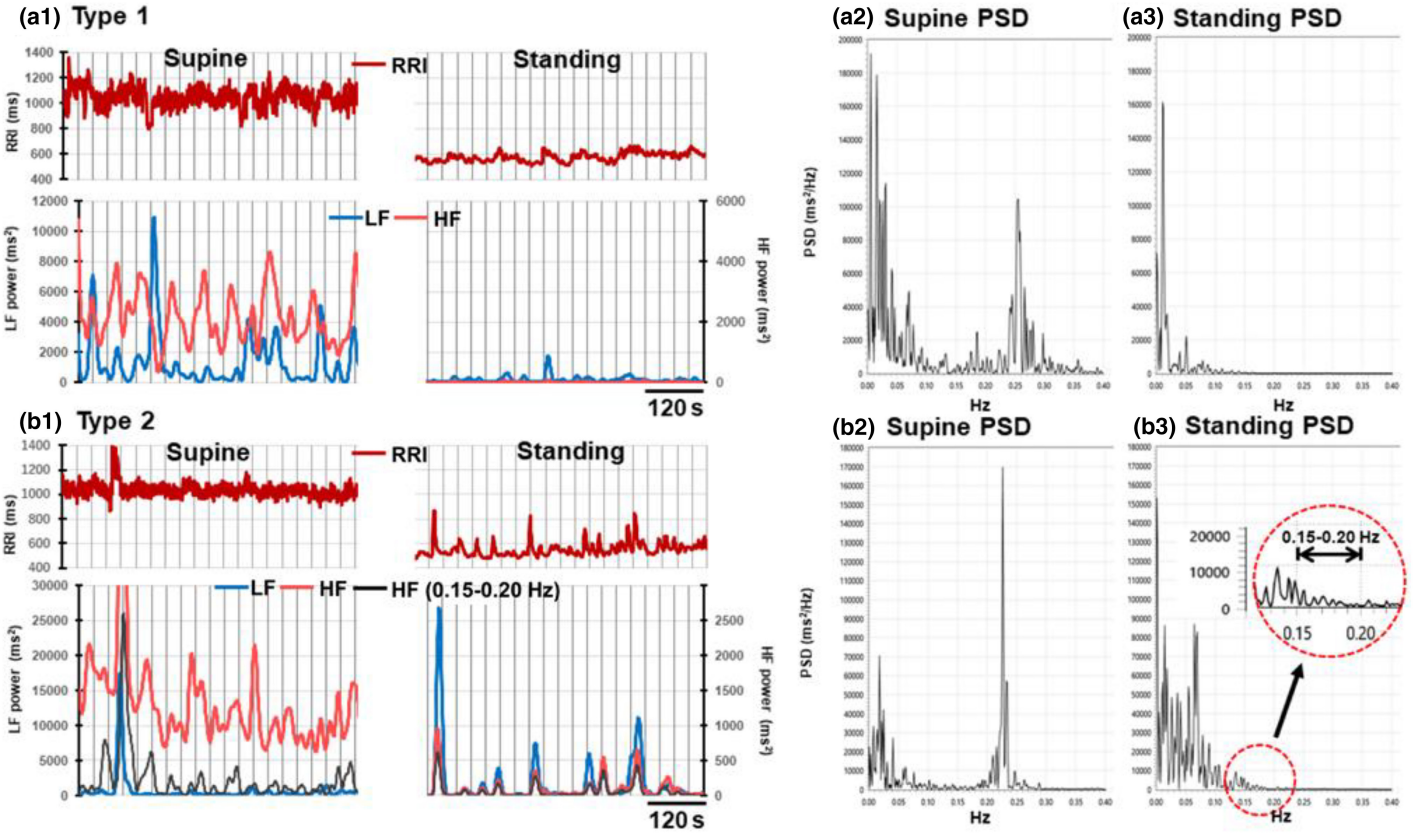
Two typical types of changes in the R‐R interval (RRI) and LF and HF powers that occurred upon standing from the supine positions. The RRI time series in the supine position and during standing each for 600 s, and the time course of changes in the LF and HF powers obtained by spectral analysis of 30‐s RRI data segments consecutively with a 1‐s shift are shown for type 1 (a1) and type 2 (b1) cases. The power spectral densities (PSDs) for these 600‐s RRI time series in the supine position and during standing are shown in (a2) and (a3) for the type 1 case and (b2) and (b3) for the type 2 case, respectively. (a1)–(a3): A typical type 1 response to the postural change from supine to standing. Both HF and LF powers were markedly reduced upon standing. (b1)–(b3): A typical type 2 response to postural change. Although the HF power was markedly reduced upon standing as in (a), the LF power increased. The PSDs (b2) in the supine position and (b3) during standing indicate that although the HF power generated by respiratory sinus arrhythmia (RSA) was almost completely suppressed during standing, there remained small but significant HF power due to the overspill of the tail of the LF component, which is shown by the enlargement of the area encircled by a red dotted line in (b3). In the bottom panel of (b1), the time course of the change in the power between 0.15 and 0.20 Hz was added as a black line. Note that the value of the HF power on the right vertical axis in the bottom panel in (b1) was reduced to 1/10 compared to that of the LF power on the left vertical axis to express the HF power increase by 10 times magnification. These results were obtained by reanalyzing the RRI time series data partially presented in Figure 4a in our previous paper (Yokobori et al., 2023).

Figure 2a1,b1 show the time courses of changes in the LF and HF powers analyzed using 30‐s segment data from each 600‐s RRI time series in the supine position and during standing in the representative type 1 and type 2 cases. In each case, the upper panel shows the RRI time series and the lower panel shows the time course of changes in the LF and HF powers obtained by spectral analysis of 30‐s RRI data segments consecutively with a 1‐s shift. The PSDs of each 600‐s data point in the supine position and during standing in both cases are shown in Figure 2a2,a3,b2,b3.

As shown in Figure 2b1, we previously found that the transient increase in LF power during standing was always accompanied by a small but clear increase in HF power in type 2 cases (see the HF power oscillations shown in orange during standing). We suggested that this HF power could originate from the vagal nerve activity that generates RSA (Yokobori et al., 2023). However, this notion should be corrected for the following reasons. First, the PSDs in Figure 2b2,b3 show that the HF power oscillation due to RSA was almost completely suppressed during standing. Second, even if a residual RSA component existed, its oscillation rhythm would not temporally coincide with the LF power increase associated with the occasional occurrence of the transient RRI increase.

Regarding the origin of this HF power increase during standing, we noted that the tail of the LF component during standing spilled over into the HF range, shown in the area encircled by a red dotted line in Figure 2b3. We calculated the ratio of the HF power in the range between 0.15 and 0.20 Hz relative to the power of the entire HF range (0.15–0.4 Hz) during standing. This ratio was 0.491 ± 0.059 (mean ± SD, *n* = 6) during standing, whereas it was 0.165 ± 0.103 (*n* = 6) in the supine position, with the former being significantly larger (*p* = 0.0002). Furthermore, we detected a significant increase in power in the frequency range between 0.15 and 0.20 Hz, which was tightly associated with the LF power increase during standing. In Figure 2b1, we added the time course of this increase in the power of the HF component between 0.15 and 0.20 Hz (line in black in the bottom trace) to those of changes in the LF and HF powers.

**FIGURE 3:**
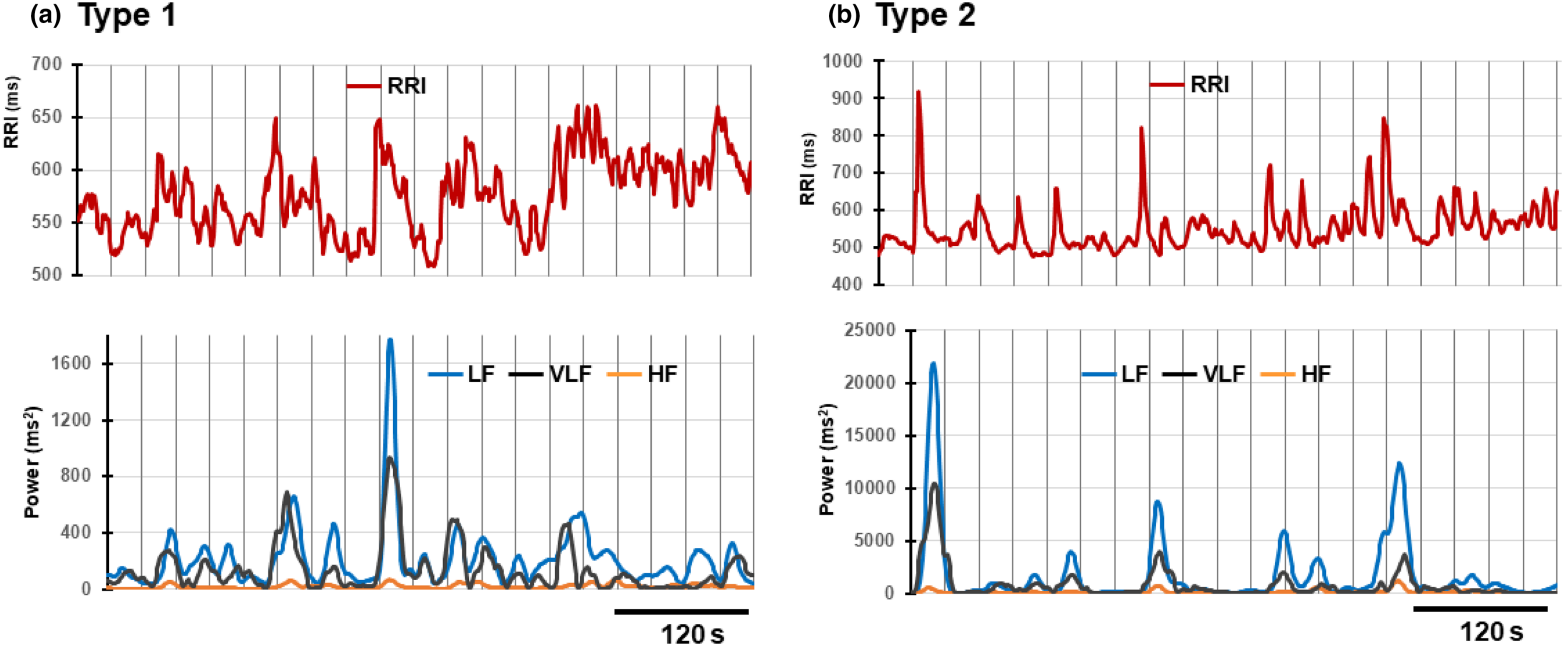
Simultaneous increases in VLF and LF powers associated with transient RRI increases in the 600‐s RRI time series data during standing. The time courses of the VLF power increase and its temporal relationship with the LF power increase linked to the transient RRI increase in type 1 (a) and type 2 (b) cases were tracked continuously by connecting the magnitudes of the powers calculated by spectral analyses of 30‐s data segments, each conducted consecutively with a 1‐s shift. The time course of the HF power change is also shown.

In the typical type 1 case shown in Figure 2a, the increase in the LF power associated with the transient RRI increase during standing was very modest. Nevertheless, we detected a small increase in the HF power linked to the increase in LF power, as shown in the following figure (Figure 3a). Furthermore, the HF component's power ratio between 0.15 and 0.20 Hz relative to the power of the whole HF range (0.15–0.4 Hz) was also larger during standing than in the supine position. This ratio was 0.345±0.155 (*n* = 6) during standing and 0.139±0.048 (*n* = 6) in the supine position, with the former being significantly larger (*p* = 0.014). These results indicate that a small increase in HF power is associated with the transient RRI increase mainly due to the overspill of lower frequency components into the HF range higher than 0.15 Hz.

### 3.2 Simultaneous increase in VLF power with LF power increase associated with transient increase in RRI

The PSDs of the RRI time series during standing shown in Figure 2a3,b3 suggested that the boundary between the LF range (0.04–0.15 Hz) and VLF range (0.003–0.04 Hz) is even more unclear than that between the LF and HF ranges, and the power increase in the VLF component would also be associated with the transient RRI increase. To examine this possibility, we analyzed the 600‐s RRI time series data starting 60 s after the postural change to standing in the typical type 1 and 2 cases shown in Figure 3. In the 30‐s RRI data segment, we tracked the time course of changes in the VLF power and their temporal relationship with the LF and HF power increases linked to the transient RRI increase in Figure 3. The results indicated that the VLF power increase was associated with the LF power increase linked to the transient RRI increase on a much larger scale than the HF power increase in both type 1 and type 2 cases.

Regarding the degree of involvement of the VLF component, the results shown in Figure 3 suggested that the magnitude of the VLF power relative to the LF power was greater in the type 1 case than in the type 2 case. To evaluate this issue quantitatively, we adopted the method described in the reanalysis section of the previous data in Materials and Methods. Briefly, to track the temporal relationship of changes in the LF and VLF powers with the transient changes in RRI, we analyzed the 30‐s data segments of the 600‐s RRI time series consecutively with a 1‐s shift, and the magnitude of each power thus obtained was tracked. Since the values of the VLF and LF power increases were obtained in 571 30‐s segments in this procedure, we calculated the means of these values to quantitatively evaluate the involvement of the VLF component relative to that of the LF component. The ratio of the mean of the VLF power relative to that of the LF power thus obtained was 0.672 ± 0.054 (*n* = 6) in the type 1 case and 0.373 ± 0.065 (*n* = 6) in the type 2 case, being significantly larger in the former case (*p* = 5.7 × 10^−6^).

In the above quantitative evaluation of the VLF component, however, there was a problem; that is, the analysis of short RRI segments such as 30 s would fail to correctly estimate the magnitude of the power of the low‐frequency range. As described in Materials and Methods, to ensure 0.0048 Hz frequency resolution, the time window for FFT processing was set to 204.8 s in the present analysis. When the RRI data length had to be reduced to identify the temporal relationship between the rapid change in RRI and changes in the powers of each frequency component, zero padding was needed to the original data to make the data number 2048. Because padded zeros do not add any new information about the magnitude of RRI increase in the lower frequency range to the original signal to properly evaluate the magnitude of changes, particularly in the power of the VLF component, it was necessary to use longer data segments. Therefore, in the next section, we conducted a series of analyses to clarify how the length of the data segment to be analyzed affects the detection of the changes in the magnitude of powers in each frequency component.

### 3.3 Effects of changing the length of the RRI data segment on detecting increases in the powers associated with transient increases in RRI in different frequency ranges

**FIGURE 4:**
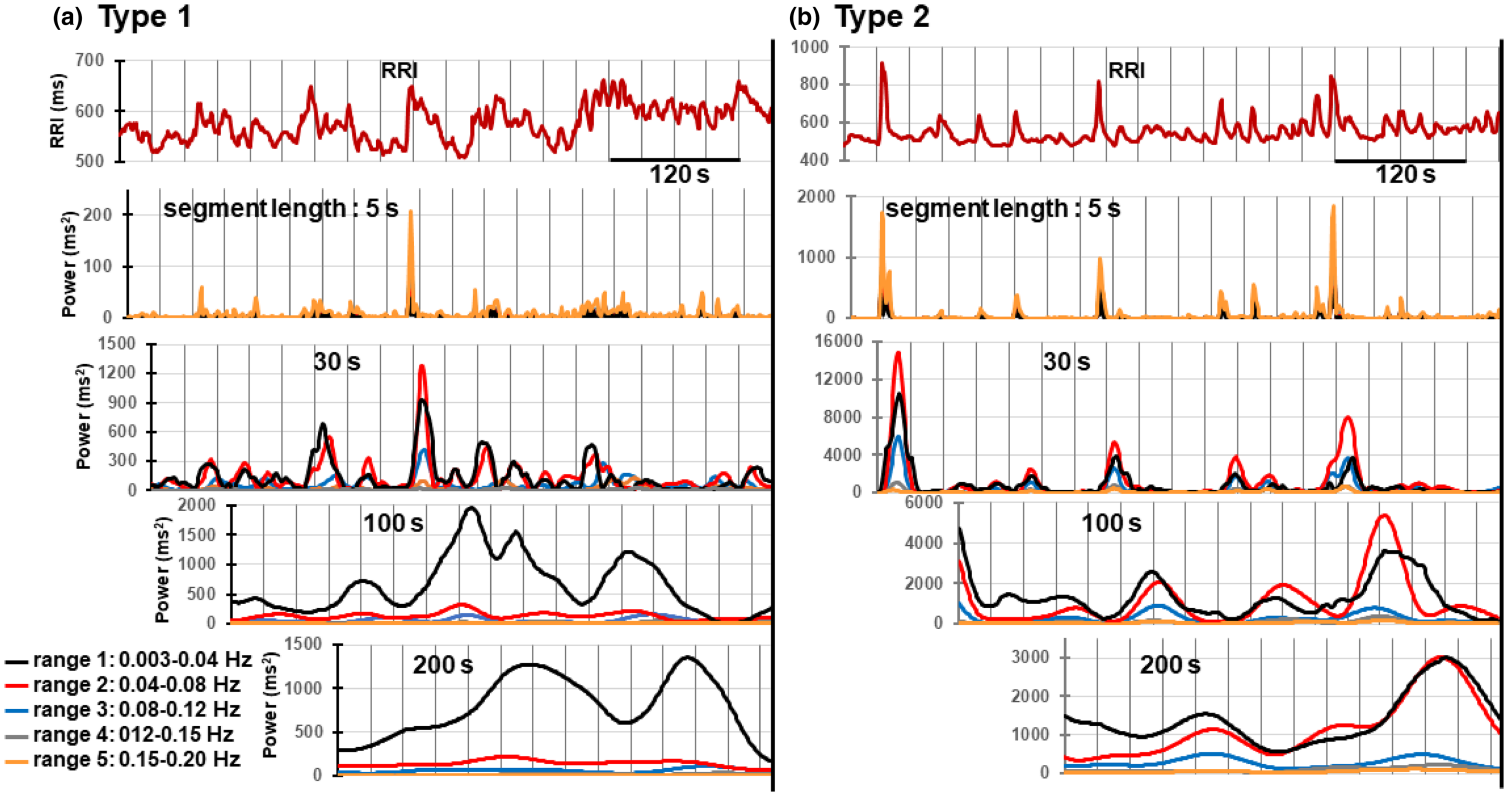
Effects of changing the length of the data segment on the detection of power increases associated with transient RRI increase in five different frequency ranges: range 1 (0.003–0.04 Hz), range 2 (0.04–0.08 Hz), range 3 (0.08–0.12 Hz), range 4 (0.12–0.15 Hz), and range 5 (0.15–0.20 Hz). Type 1 (a) and type 2 (b) 600‐s RRI time series during standing (the first panels in (a) and (b)) shown in Figure 2 were analyzed. The second to fifth panels in (a) and (b) show the time courses of the changes in the powers in the five frequency ranges, which were obtained by connecting the magnitudes of the powers calculated by spectral analyses of 5‐s, 30‐s, 100‐s, and 200‐s data segments, each conducted consecutively with a 1‐s shift.

Figure 4 shows how changing the length of the RRI data segment to be analyzed affects the detection of changes in the time course and magnitude of powers in various frequency ranges. In this figure, we conducted the spectral analysis of 5, 30, 100, and 200‐s data segments of the 600‐s RRI time series during standing in type 1 and type 2 cases shown in Figure 2. A segment length of 200 s was selected to minimize zero padding and was used as a control to check the influence of zero padding. The frequency range was divided into five: range 1 (0.003–0.04 Hz), range 2 (0.04–0.08 Hz), range 3 (0.08–0.12 Hz), range 4 (0.12–0.15 Hz), and range 5 (0.15–0.20 Hz). Range 1 is the VLF range, and range 5 is the lower frequency region of the HF band. The LF band was divided into three ranges, 2, 3, and 4, to roughly equalize the width of each range. The spectral analysis was performed consecutively with a 1‐s shift, and the magnitudes of powers in the five frequency ranges thus obtained with a 1‐s shift were connected and are shown in Figure 4. The means of the magnitudes of powers obtained with a 1‐s shift were used to quantitatively compare the magnitudes of powers obtained in each frequency range in Table 1.

**TABLE 1:** Increases in the powers of the components of five frequency ranges in the 600‐s RRI time series estimated by changing thedata segment length from 5 s to 600 s.

Frequency range	Type	Segment length				
		5 seconds	30 seconds	100 seconds	200 seconds	600 seconds
Range 1 0003–0.04 Hz	1	0.005±0.003	0.184±0.056	0.554±0.101	0.669±0.050	0.728±0.044
	2	0.006±0.001	0.205±0.062	0.347±0.094	0.397±0.108	0.387±0.067
Range 2 0.04–0.08 Hz	1	0.006±0.003	0.165±0.047	0.130±0.041	0.128±0.041	0.146±0.036
	2	0.009±0.001	0.318±0.069	0.341±0.077	0.361±0.085	0.375±0.085
Range 3 0.08–0.12 Hz	1	0.008±0.004	0.079±0.016	0.084±0.021	0.084±0.025	0.088±0.030
	2	0.013±0.001	0.181±0.024	0.165±0.049	0.164±0.049	0.174±0.064
Range 4 0.12–0.15 Hz	1	0.008±0.004	0.026±0.014	0.020±0.009	0.019±0.007	0.019±0.007
	2	0.012±0.001	0.043±0.005	0.035±0.010	0.035±0.013	0.038±0.022
Range 5 0.15–0.20 Hz	1	0.015±0.007	0.020±0.017	0.018±0.016	0.018±0.016	0.020±0.017
	2	0.023±0.003	0.022±0.008	0.021±0.003	0.022±0.002	0.026±0.005

*Note*: Values are means ± SD; *n* = 6. The absolute value of each power was normalized using the total power obtained from a 600‐second segment length in both type 1 and type 2 cases.

The main observations shown in Figure 4 are as follows:

1. In the 5‐s segment analysis, a sharp increase in the power in range 5 was detected almost simultaneously at the onset of the transient increase in RRI in both type 1 and type 2 cases. This power increase decreased at the lower frequency range, minimal in range 1.
2. In the 30‐s segment analysis, increases in the powers associated with the transient increase in RRI occurred mainly in ranges 1, 2, and 3 (VLF and lower frequency region of the LF range). A small increase was detected in ranges 4 and 5.
3. In the 100‐s segment analysis, an increase in the power in range 1 became prominent in the type 1 case. This change also occurred in the type 2 case, but it was less prominent compared to the type 1 case. In this segment length, a direct relationship between individual power increase and each transient increase in RRI was lost in all frequency ranges due to the deterioration of time resolution. However, the temporal changes in a group of RRI increase events were reflected in a group of delayed power increases in each frequency range.
4. In the 200‐s segment analysis, a further increase in the power in range 1 continued in both type 1 and type 2 cases. In this segment length, the magnitude of the powers in all frequency ranges became similar to those obtained from the spectral analysis of the entire 600‐s RRI time series during active standing. In the following description, the powers obtained from the spectral analysis of the entire 600‐s RRI time series data are treated as the data obtained by 600‐s segment analysis.

Regarding the effects of changing the length of the RRI data segment, similar results as shown in Figure 4 were obtained without exception in all type 1 and type 2 cases. However, there was a large dispersion in the absolute values of these powers among the six RRI time series data in both type 1 and type 2 cases. This dispersion was due to the large difference in the total power of the range 1–5 among each of the six measurements in both type 1 and 2 cases. Therefore, we normalized each value by the total power obtained with a 600‐s segment length and presented the results of the calculation in Table 1 to show the differences in the mean powers in the five frequency ranges estimated in the segments of the five different lengths in six type 1 and type 2 cases.

**FIGURE 5:**
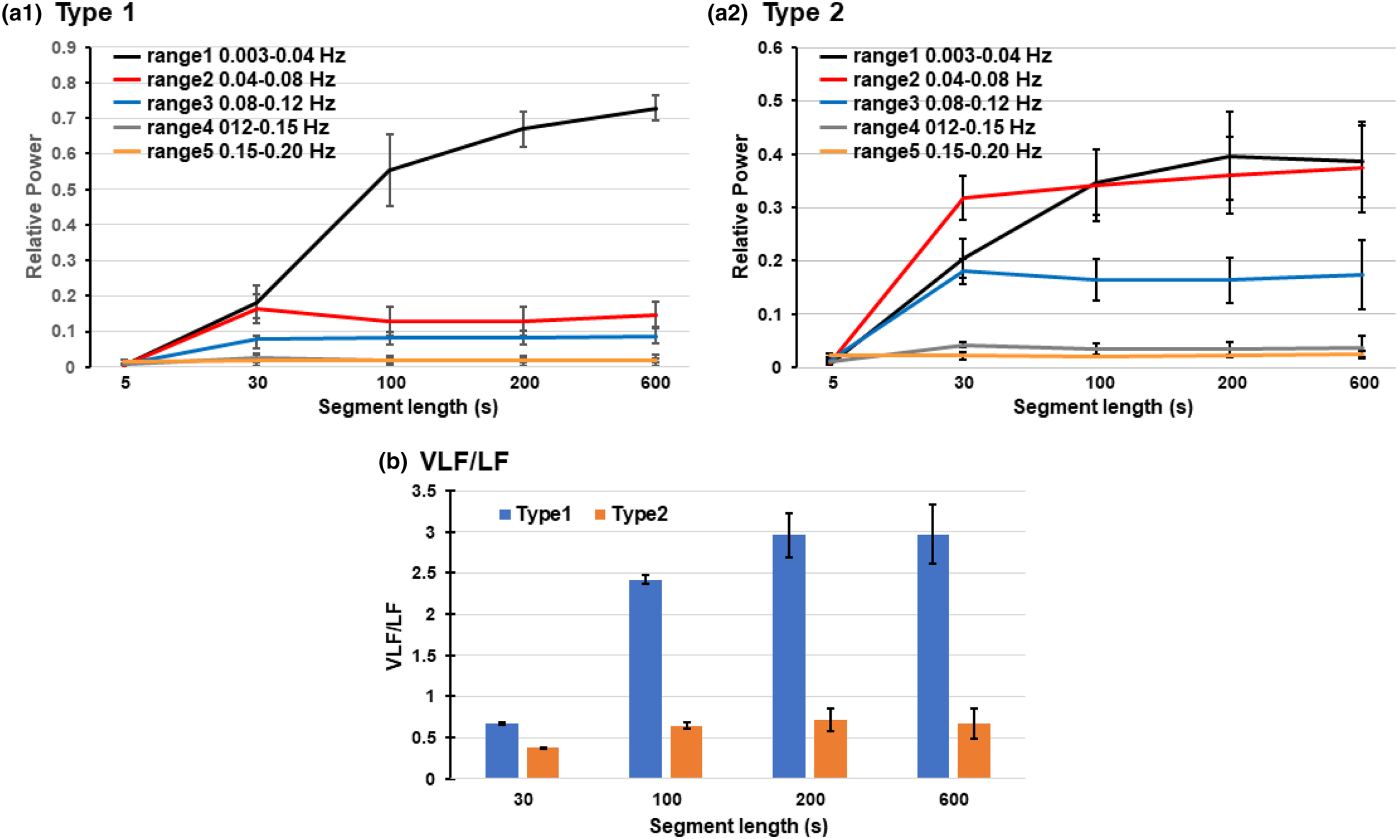
(a1) and (a2) Graphs to summarize the effects of changing the data segment length in the 600‐s RRI time series during standing on the estimation of power increases in five different frequency ranges in type 1 (a1) and type 2 (a2) cases. The average values of six type 1 and six type 2 cases are plotted as the mean ± standard deviation (SD). Each value of the power magnitude was normalized by the total power obtained in the 600‐s segment. (b) Effects of changing the data segment length on the ratio of VLF power relative to LF power. The LF power was obtained as the sum of powers in ranges 2–4. This ratio in the type 1 case markedly increased when the segment length was increased to 100 s, and it reached a level similar to that of the 600‐s segment at the 200‐s segment. The ratio was significantly larger in the type 1 case than in the type 2 case for all segment lengths (*p* < 2.1×10^−4^).

Figure 5 summarizes the main results obtained from the analyses in this section. First, graphs (a1) and (a2), which show the results of type 1 and type 2 cases, respectively, indicate that the powers in ranges 1–4 increased as the length of the segment was increased from 5 s to 30 s. When the segment length was further increased to 200 s, the power in range 1 (VLF range) continued to increase in type 1 and type 2 cases. In contrast, the power in range 2–4, the sum of which was the power of the LF component, was not significantly affected by increasing the segment length from 30 s to 600 s. Second, in graph (b), the ratio of the VLF power relative to the LF power was significantly larger in the type 1 case than in the type 2 case, irrespective of the segment length (*p* < 2.1×10^−4^ in all segment lengths) because the LF power was much greater in the type 2 case. Furthermore, this ratio in the type 1 case markedly increased as the segment length increased to over 100 s. At 600‐s segment length, the ratio was 2.971±0.681 (*n* = 6) in the type 1 case and 0.675±0.188 (*n* = 6) in the type 2 case.

### 3.4 Physiological correlate of simultaneous increases in the powers of VLF, LF, and HF components associated with transient increases in RRI

Next, we attempted to identify the origin and physiological correlate of the simultaneous increases in the powers of the VLF, LF, and HF components. Several lines of evidence have indicated a close relationship between LF fluctuation in HRV and arterial baroreceptor reflex (Cevese et al., 2001; Goldstein et al., 2011; Grasso et al., 1997; Moak et al., 2007; Rahman et al., 2011; Sleigh et al., 1995).

In this study, we examined the relationship between changes in the beat‐to‐beat arterial blood pressure and the simultaneous increases in the power of VLF, LF, and HF components. We conducted 16 measurements in eight subjects, of which 11 were type 1 cases and five were type 2 cases. In these measurements, the subjects wore both the beat‐to‐beat finger artery blood pressure monitoring device and the electrocardiogram sensing device, which allowed simultaneous recordings of the time series of arterial blood pressure, IBI, and RRI.

**FIGURE 6:**
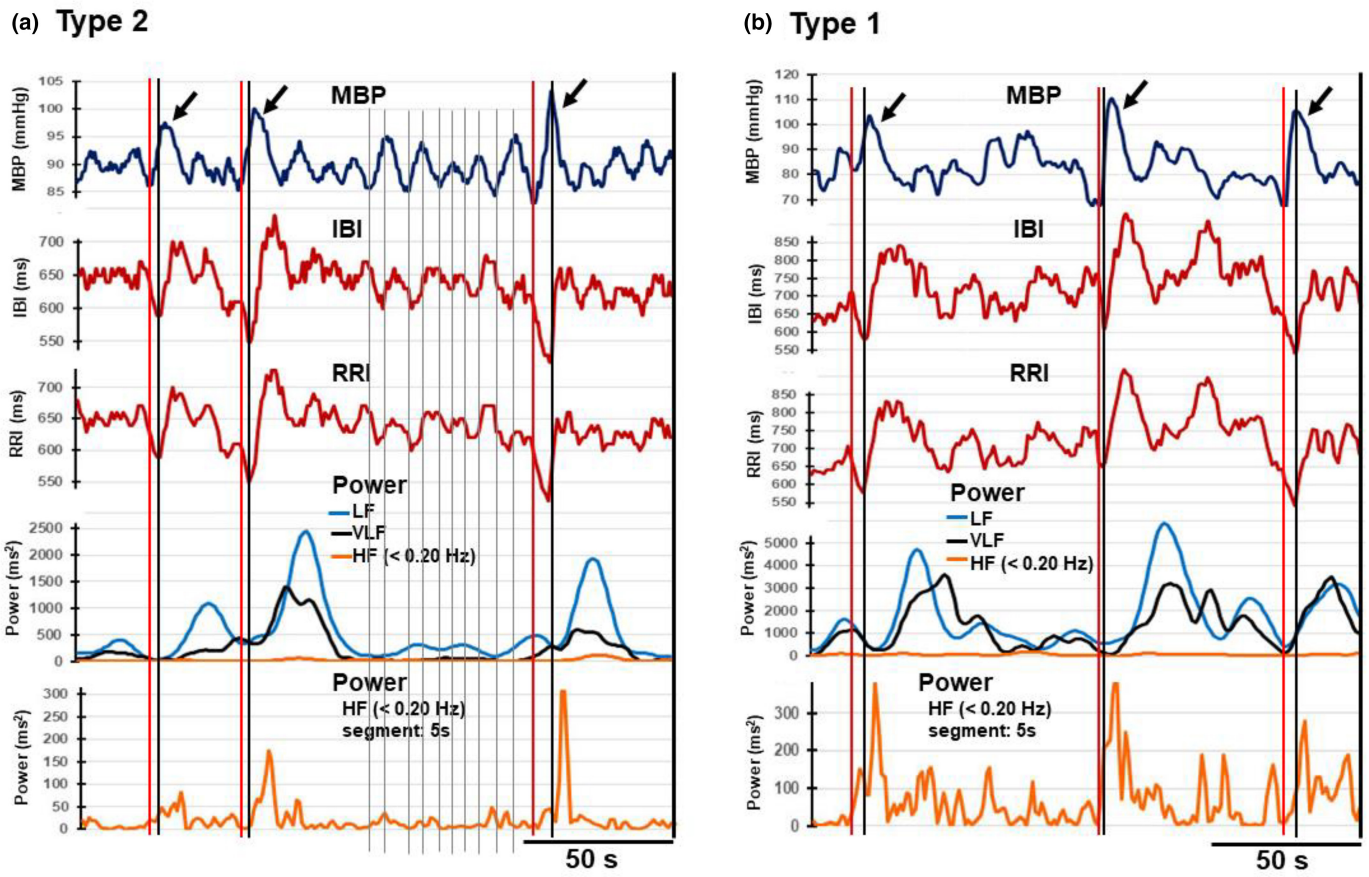
Simultaneous recordings of arterial blood pressure, interbeat interval (IBI), RRI, and changes in VLF, LF, and HF (0.15–0.20 Hz range) powers in type 2 (a) and type 1 (b) cases. In both (a) and (b), the top (first) panel shows the time series of arterial mean blood pressure (MBP) changes, the second panel shows that of IBI estimated by the time point of the systolic blood pressure, and the third panel shows the RRI time series. The time courses of IBI and RRI were similar. The fourth panel shows the time course of changes in VLF, LF, and HF power obtained by the 30‐s segment analysis of the RRI time series shown in the third panel. The bottom (fifth) panel shows the time course of change in HF power obtained by the 5‐s segment analysis. Three black arrows in the top panels in both (a) and (b) show the occurrence of higher blood pressure increases with a faster rate of rise than other blood pressure increases. These larger pressure increases initially caused a decrease in IBI (RRI), followed by a sudden reversal to the sharp increase. Three pairs of red and black vertical lines are drawn from the top to the bottom panel at the black arrows. The red line indicates the onset of the MBP rise, and the black indicates the time when the abrupt reversal of IBI (RRI) from the negative to the positive direction occurred. These three pairs of red and black lines in type 1 and type 2 cases show that the clear simultaneous increase in VLF, LF, and HF powers was preceded by this rapid reversal of IBI (RRI). In contrast, 10 gray vertical lines drawn between the second and third arrows in (a) show that when smaller MBP rises occurred without induction of the abrupt reversal of IBI (RRI), they failed to induce the clear simultaneous VLF and LF power increases, although the onsets of the blood pressure rise and fall temporally coincided with those of IBI (RRI) decrease and increase, respectively.

Figure 6a,b show the results in a representative type 2 case (standing: supine ratio of the LF powers = 2.12) and type 1 case (standing: supine ratio of the LF powers = 0.36), respectively. In both Figure 6a,b, five panels from the top to the bottom are shown. The top (first) panel shows temporal changes in the mean arterial blood pressure (MBP) during a period of standing, the second panel shows those in the IBI, the third panel shows those in the RRI, the fourth panel shows those in the VLF, LF, and HF (0.15–0.2 Hz range) powers obtained from 30‐s RRI data segments, and the bottom (fifth) panel shows those in the HF (0.15–0.2 Hz range) power obtained from 5‐s data segments. As described in Figure 4, reducing the segment length from 30 s to 5 s improved the time resolution of the spectral analysis. It helped detect the onset of the HF power increase in the RRI time series. The IBI time series was similar to that of the RRI time series, and the results of spectral analyses of both time series were identical.

In Figure 6a, we found vasomotor waves (~0.1 Hz) in the continuous recordings of MBP in the first panel. In these blood pressure oscillations, typically indicated by gray vertical lines between the second and third arrows, the onsets of the MBP rise and fall temporally coincided with those of IBI (RRI) decrease and increase, respectively, which would be at least partly due to negative feedback arterial baroreceptor reflex mechanisms (Hall & Hall, 2021; Julien, 2006). Interestingly, as shown by the three arrows in the first panel, when a larger MBP increase with a faster rate of rise than in other oscillations occurred, an initial decrease in IBI was abruptly converted to a rapid increase in a few seconds (2–6 s in most cases). The MBP started to return to the original level during the rapid IBI increase following this reversal of the direction of IBI change. Throughout the top‐to‐bottom panels in this figure, three pairs of red and black vertical lines were drawn to indicate the onset of the sharp MBP rise and the time point when the change in the IBI in the initial negative direction abruptly reversed to the positive direction, respectively. In the fourth panel, the spectral analysis of the 30‐s RRI data segment showed that the LF power increase accompanied by the VLF power increase appeared around the time of the abrupt reversal of the direction of the change in IBI. Because the time resolution of the result obtained by 30‐s segment analysis in the fourth panel was not sufficient to specify the time point of the onset of increases in VLF, LF, and HF powers, we added the fifth panel showing the change in HF power (0.15–0.2 Hz range) obtained by 5‐s segment analysis. This panel showed that the increase in HF power occurred clearly at the abrupt reversal of the direction of the IBI change.

In the type 1 case shown in Figure 6b, we could not identify clear vasomotor waves in the MBP time series. Slow oscillations comparable to vasomotor waves were less regular, and small fluctuations of ~20 Hz occurred frequently. However, larger MBP increases with a faster rate of rise than in other types of fluctuations indicated by the three arrows also caused similar events as seen in the type 2 case. Namely, as indicated by three pairs of red and black vertical lines, the MBP increase with a fast rate of rise caused an initial decrease in the IBI, which rapidly reversed to a steep increase. The VLF, LF, and HF power increase occurred associated with the abrupt reversal of the IBI change from negative to positive. The bottom panel shows the change in HF power (0.15–0.2 Hz range) obtained by 5‐s segment analysis, which revealed that a clear increase in HF power occurred at the time point of the abrupt reversal of the direction of the IBI change. However, smaller HF power increases, which coincided temporally with the onset of either a decrease or increase in the IBI, were detected frequently in the type 2 case. Figure 6b also shows that the involvement of the VLF power increase relative to that of the LF power is greater in the type 1 case than in the type 2 case.

Throughout these experiments, we noted that the abrupt change of IBI (RRI) from the negative to positive direction induced by the transient blood pressure increase often occurred before it reached the peak, as clearly shown in the first and second events in Figure 6b. This finding suggests that among the arterial blood pressure oscillations, the determinant of whether the baroreceptor reflex pathway responsible for the abrupt reversal of the direction of IBI (RRI) change is activated or not is the rate of rise of the blood pressure increase rather than its change in amplitude.

Based on these results, we conclude that the simultaneous increase in the VLF, LF, and HF powers associated with the transient RRI increase is generated by the arterial baroreceptor reflex in response to the rapid rise in arterial blood pressure in both type 1 and type 2 cases. In addition, there could be two different regulatory mechanisms in the arterial baroreceptor reflex. One is tonic regulation, which functions in lower levels of blood pressure oscillations, and the other is more dynamic regulation for greater pressure changes that occur rapidly. Increases in the VLF and LF powers would be generated when the latter regulatory mechanism operates (see Discussion).

## 4 DISCUSSION

In this study, we found that the transient increase in RRI that occasionally occurred during standing was associated with simultaneous increases in the powers of VLF, LF, and HF components and that these changes originated from the arterial baroreceptor reflex in response to transient rapid rise in blood pressure. Here, we discuss several issues related to these two main findings.

### 4.1 Method to deal with the trade‐off relationship between time and frequency resolutions in spectral analysis in HRV studies

The autonomic nervous system plays a pivotal role in short‐term cardiovascular control on a time scale of seconds to minutes. It is evident that the conventional standard method, which examines stationary 300‐s blocks of RRI data, has severe limitations when analyzing the function of this system with spectral analysis of HRV. It does not allow the detection of temporal localization of dynamic changes in the RRI sequence. In this study, we performed the time‐frequency analysis based on STFT to elucidate dynamic changes in the spectral powers linked to transient RRI increases that sporadically occur during active standing. In this analysis, the PSD of a short time data sequence with a discrete length is consecutively obtained with STFT. The time sequence of the PSDs thus obtained provides a valuable visualization of how time‐domain signals change in terms of frequency components. To deal with the trade‐off relationship between time and frequency resolutions, which is an inevitable restriction in signal processing using Fourier transform, we attempted to analyze the RRI time series data after splitting it into shorter segments from 5 s to 200 s. We performed spectral analysis in each segment consecutively with a 1‐s shift.

In the 5‐s segment analysis, a sharp increase in the power in range 5 (0.15–0.2 Hz) occurred almost simultaneously at the onset of the transient increase in RRI. This transient RRI increase corresponds to the HF power increase detected by the 30‐s segment analysis shown in Figure 2b1 and the PSD in Figure 2b3. The power increase with the same time course was also detected in ranges 1–4. However, the magnitude was the largest in range 5, and it gradually decreased in the lower frequency range, being minimal in range 1 without exception in all 12 cases tested. It is most likely that the sharp power increases detected in ranges other than range 5 were sidelobes caused by the overuse of zero padding. Thus, the result of the 5‐s segment analysis contained artifacts. Nevertheless, it was still helpful to detect the timing of the onset of changes in the power induced by the transient RRI increase.

In the 30‐s segment analysis, we were able to identify the changes in the powers of the VLF, LF, and HF components related to each transient RRI increase and also estimate the degree of changes in their magnitudes roughly. Therefore, the 30‐s segment analysis was the most useful for understanding the relationship between transient RRI changes and corresponding spectral power changes. However, it was less capable than the 5‐s segment analysis in precisely detecting the timing of the onset of the spectral power change and less capable than the 200‐s segment analysis in quantitatively evaluating the magnitude of the power increase in the VLF range.

In the 100‐s segment analysis, a direct relationship between individual power increase and each transient increase in RRI was lost in all frequency ranges due to the deterioration of time resolution. Nevertheless, we detected with this 100‐s segment analysis that an increase in the power in range 1 became prominent in the type 1 case, and this increase also occurred in the type 2 case. Next, in the 200‐s segment analysis, a further increase in the power in range 1 continued in both type 1 and type 2 cases. In this segment length, the magnitude of the powers in all frequency ranges became almost similar to those obtained from the spectral analysis of the entire 600‐s RRI series data during standing.

Thus, we could obtain the whole picture of the changes in power linked to the transient RRI change by integrating the results of spectral analyses of 5‐s to 200‐s segments. This approach would be useful as a practical method for various HRV studies to elucidate the properties of dynamic changes in the RRI time series. However, a critical problem with this method is that although the proper selection of the length of the data segment is crucial for obtaining valuable information, it has to be done manually depending on the specific requirements of the data analysis. It is desirable to establish a method that automatically provides optimum data segment length (see Limitations).

### 4.2 Relationships between simultaneous increases in the powers of VLF, LF, and HF components and arterial baroreceptor reflex

The most interesting finding in this study was that among naturally occurring arterial blood pressure oscillations, a steep rise in arterial blood pressure initially decreased RRI, but in a few seconds, the direction of the RRI change was reversed from decrease to steep increase. This abrupt reversal of the direction of the RRI change preceded the simultaneous increases in the powers of VLF, and LF components. Because a smaller blood pressure rise neither induced the abrupt reversal of the direction of the RRI change nor the clear increase in the VLF and LF powers linked to the transient RRI increase, we suggested that there could be two different types of regulatory mechanisms in the arterial baroreceptor reflex. Regarding this issue, it is known that there are two types of arterial baroreceptor afferents, larger diameter A‐fiber afferents (type 1) and smaller A‐fiber/non‐myelinated C‐fiber afferents (type 2), in various animals (rats, rabbits, dogs) and humans (Porzionato et al., 2019; Sturdy et al., 2017). Type 1 afferents have a distinct threshold pressure below which they do not fire and have a relatively large minimum firing rate. In contrast, type 2 afferents are active in almost all pressure ranges, although they have a pressure threshold at which their firing rate increases in response to a rise in blood pressure. The firing rate is higher in type 1 afferents than in type 2 afferents (Porzionato et al., 2019; Seagard et al., 1990; Sturdy et al., 2017). To clarify the differential functional role of these two types of baroreceptor afferents, Seagard et al. (1993) selectively eliminated the activities of these two types of afferents by anodal and anesthetic block in dogs. The results of their experiments have indicated that the sudden‐onset, high‐frequency discharges at high thresholds in type 1 baroreceptor afferents contribute to the regulation of dynamic pressure changes and can be the primary buffer to prevent sudden changes in arterial blood pressure. In contrast, continuous firing patterns of type 2 afferents marked by low frequencies and wide operating ranges are more suitable for tonic regulation of baseline resting levels of blood pressure. These findings suggest that the abrupt reversal of the RRI change from the negative to positive direction induced by a rapid rise in blood pressure shown in the present study is due to the activation of type 1 baroreceptor afferents.

Taylor et al. (1998) have shown that normal subjects have a substantial RRI spectral power in the VLF range (0.003–0.03 Hz) in both the supine and 40‐degree upright tilt positions and that this VLF power, as well as the LF and HF powers, is almost completely abolished by cholinergic blockade (Hayano et al., 1991; Jokkel et al., 1995; Taylor et al., 1998). Regarding the origin of the RRI power in the VLF range, however, Taylor et al. (1998) concluded that it was not mediated by the arterial baroreflex mechanism because their estimates of coherence between the arterial systolic blood pressure (SBP) and RRI time series at VLF were not statistically significant. Cevese et al. (2001) also performed spectral and cross‐spectral analyses of the time series of RRI and SBP oscillations in normal subjects in the supine position with metronome respiration at 0.25 Hz. They found a clear peak of LF (~0.1 Hz) power increase in the time series of RRI and SBP and a high coherence between them with an average phase shift of −65.1 degree (1–2 heartbeat delay of RRI to SBP in the time domain) in control conditions. They also found that when the subjects were administered an alpha‐adrenergic blocker to inhibit fluctuations in arterial blood pressure along with angiotensin II to maintain the MBP at control levels, RRI LF power fluctuations were almost completely abolished. They, therefore, concluded that an arterial baroreflex mechanism almost entirely accounts for the oscillation of RRI in the LF range. Regarding the involvement of VLF powers, they found that when the alpha‐adrenergic blockade abolished RRI LF power fluctuations, other peaks of the RRI and SBP powers appeared at a lower frequency range named reduced low frequency (rLF) range, which was in the lower part of, or just below the range generally classified as the LF range. In this range, high coherence between RRI and SBP was retained. Although the mechanism(s) underlying this shift of the peaks of the RRI and SBP powers caused by the alpha‐adrenergic blockade is presently unknown, we speculate that the decrease in the degree of SBP oscillation in both its amplitude and rate of rise would be one of the causes of this shift.

Although our present results are not completely incompatible with those reported by Cevese et al. (2001), the details differ. For example, if our conclusion is correct, a significant coherence between RRI and SBP in the VLF range would be expected even in control conditions without alpha‐adrenergic blockade. Possible reasons for these discrepancies are as follows. First, we limited our analysis to the RRI time series linked to the transient RRI increase that occurred only occasionally during active standing. In contrast, the time series of RRI and SBP oscillations as the target of the spectral and cross‐spectral analyses would contain various arterial pressure oscillations unrelated to the transient RRI increase. Second, the experiments by Taylors et al. and Cevese et al. were performed mainly in subjects in the supine position. During this series of experiments, we noted that in the supine position, the waveforms of the RRI time series are much more complex than those during standing. The reason for our avoiding the analysis of the RRI record in the supine position was that the complex waveforms of the RRI time series hindered the precise detection of the transient increase in RRI. The possibility that RRI and SBP oscillations not linked to the transient RRI increase are contained in the cross‐spectral analysis would increase in the supine position. Third, we have also noted that in addition to the marked RSA‐related HF oscillations, the fluctuations of powers in the wide frequency ranges are accentuated in the supine position.

In future studies, it is essential to systematically examine differences in the changes in the powers in the wide frequency ranges linked to the transient RRI increase between in the supine position and during standing, and to elucidate the mechanisms underlying these differences.

### 4.3 Limitations

In this study, we analyzed nonstationary RRI data signals with the widely‐used time‐frequency analysis method based on STFT. We found that the transient RRI increase is linked to the simultaneous increases in the powers of VLF, LF, and HF components, and its physiological correlate is the arterial baroreceptor reflex. However, there is a limitation in the method adopted in this study. That is, though the proper selection of a data segment length during the calculation of the short‐term PSD is crucial for drawing useful information from the signals, it has to be selected manually according to private experiences. As a promising alternative to the present method, continuous time wavelet transform analysis can be considered since it overcomes the dilemma of conventional spectral analysis by automatically choosing a small window at high frequency and a longer window at low frequency (Acharya et al., 2006; Pichot et al., 1999; Tzabazis et al., 2018). Although the results obtained by wavelet transform analysis depend on the mother wavelet chosen for the analysis, it is worth trying to find an appropriate wavelet that yields the best results regarding clarity and distinction of the pattern in our target. In future trials, we should compare the present results with those obtained with a wavelet transform analysis.

The mechanism by which the sudden blood pressure increase with a fast rate of rise occurs sporadically, which generates the transient RRI increase, was out of the scope of this study. However, it is an interesting issue, and the origin of the sporadic changes in the cardiovascular system is actively debated. Karavaev et al. (2019) have shown that the irregularity in HRV originates not only from the stochastic influence but also from the chaotic dynamics of the control by the autonomic nervous system. Sharma (2009) describes that chaotic behavior is observed in systemic blood pressure and is suggested to be partly attributable to chaotic fluctuations in peripheral vascular resistance. As a future work, it is an attractive theme to clarify the chaotic behavior of the heart and blood vessels and create a model capable of anticipating the timing when the transient RRI increase occurs.

## 5 CONCLUSION

During active standing, we conducted spectral analysis of data segments of various lengths (5, 30, 100, and 200 s) of the RRI time series to clarify the relationships among transient changes in the RRI and VLF, LF, and HF powers. The results indicated that a transient increase in the RRI that occurred occasionally during standing was associated with simultaneous increases in the powers of the VLF, LF, and HF components. To identify the temporal relationships between individual transient increases in RRI and the resulting spectral power change, analysis of the RRI data segment shorter than 30 s was needed, whereas a segment of 200‐s was needed to properly evaluate the magnitude of the increases in the VLF power. Monitoring beat‐to‐beat blood pressure, we found that the arterial baroreceptor reflex caused simultaneous increases in the VLF, LF, and HF powers in response to a transient blood pressure increase with a rapid rate of rise.

## AUTHOR CONTRIBUTIONS

Seiji Ozawa, and Satoshi Mitsuyama designed the study. Satoshi Mitsuyama, Teruhiko Sakamoto, and Seiji Ozawa performed the experiments. Satoshi Mitsuyama, Teruhiko Sakamoto, Seiji Ozawa, and Toru Nagasawa analyzed the data. Seiji Ozawa, and Satoshi Mitsuyama drafted the manuscript. Toru Nagasawa, and Kazuomi Kario edited and revised the manuscript.

## CONFLICT OF INTEREST STATEMENT

The authors declare no conflicts of interest associated with this study.

## ETHICS STATEMENT

This study was approved by the Ethical Committee of Takasaki University of Health and Welfare. All subjects provided written informed consent.
